# Endovascular Management of Intracranial Atherosclerosis-Related Large Vessel Occlusion With the A Direct Aspiration First-Pass Thrombectomy Compared With Solumbra Technique

**DOI:** 10.3389/fneur.2021.643633

**Published:** 2021-03-02

**Authors:** Zhao-Shuo Li, Teng-Fei Zhou, Qiang Li, Min Guan, Huan Liu, Liang-Fu Zhu, Zi-Liang Wang, Tian-Xiao Li, Bu-Lang Gao

**Affiliations:** Henan Provincial People's Hospital, Henan University, Zhengzhou, China

**Keywords:** intracranial atherosclerosis-related, large vessel occlusion, ADAPT, Solumbra, endovascular treatment

## Abstract

**Background:** To investigate the effect of the A Direct Aspiration First-Pass Thrombectomy (ADAPT) vs. Solumbra technique in the treatment of acute intracranial atherosclerosis-related large vessel occlusion (LVO).

**Methods:** Patients with acute atherosclerosis-related LVO who had undergone endovascular treatment were retrospectively enrolled into two groups: The Solumbra and ADAPT groups. The clinical data were analyzed.

**Results:** Patients (104) were enrolled with 48 in the Solumbra and 56 in the ADAPT group. The mean time from femoral access to recanalization was significantly (*P* < 0.05) shorter in the ADAPT than in the Solumbra group. The recanalization time at the first line was significantly shorter in the ADAPT group than in the Solumbra group (17 ± 10.21 vs. 26 ± 15.55 min, *P* = 0.02). However, the rate of switching to the alternative was significantly higher in the ADAPT group than that in the Solumbra group (46.42 vs. 33.33%, *P* = 0.01). Eighty-two patients had eventual recanalization, resulting in a final recanalization rate of 78.85%. At 3-month clinical follow-up for all patients, the good prognosis rate reached 51.92% with good prognosis in 24 patients (50%) in the Solumbra and 30 (53.57%) in the ADAPT group. The rate of symptomatic intracranial hemorrhage was 18.75% (*n* = 9) in the Solumbra and 19.64% (*n* = 11) in the ADAPT group. The mortality rate was 21.15% (22/104). Among 80 (76.92%) patients who had angiographic follow-up (3–30 months), five (6.25%) patients experienced in-stent stenosis, and two (2.5%) experienced asymptomatic stent occlusion.

**Conclusion:** In patients with acute intracranial atherosclerosis-related LVO, clinical outcomes treated using the ADAPT technique are comparable with those using the Solumbra technique, and more patients need additional remedial measures if treated with the ADAPT technique.

## Background

As a significant health burden worldwide, acute ischemic stroke is a leading cause of morbidity and mortality; however, early revascularization and reperfusion are correlated with improved clinical outcomes (1, 2). For acute intracranial large vessel occlusion (LVO), mechanical thrombectomy has become a standard approach of treatment because of the high efficacy and good prognoses (3–6). However, the etiology of LVO is different in different ethnics, with intracranial atherosclerosis as the most common cause of ischemic stroke and more common in Asian, Spanish, and Afro-American populations (7, 8). The clinical presentation, risk factors, and demographic features are different between patients with acute intracranial atherosclerosis-related LVO (ICAS-LVO) and those with thromboembolism-related LVO (8). Mechanical thrombectomy with a stent retriever has become the mainstay of modern endovascular therapy for LVO caused by thromboembolism, but the stent retriever is less efficient for atherosclerosis-related LVO (9–11). Moreover, reocclusion and residual stenosis are often encountered in the endovascular management of atherosclerosis-related LVO, and rescue treatment with balloon angioplasty is frequently required for complete recanalization (9, 12, 13). Since fast recanalization is the most important factor in determining the clinical outcomes, multiple endovascular management techniques are needed to recanalize intracranial atherosclerosis-related LVO compared with thromboembolism-related LVO. Two currently principal techniques for mechanical thrombectomy are (1) application of a stent retriever like the Solitaire FR stent (Medtronic Neurovascular, Irvine, CA, USA) and (2) direct aspiration of the thrombus with the technique of A Direct Aspiration First-Pass Thrombectomy (ADAPT) using a large-bore aspiration catheter like the ACE 64 or 5 Max ACE catheter (Penumbra, Alameda, CA, USA) (14, 15). Moreover, a stent retriever can be used together with direct aspiration at the proximal end of a thrombus at the time of mechanical thrombectomy (16–18). The Solumbra technique uses the Solitaire FR stent retriever for mechanical thrombectomy in combination with proximal thrombus aspiration using the Penumbra aspiration catheter (16–18). The Solumbra technique seems better in complete removal of the thrombus because it combines both the mechanical thrombectomy with a stent retriever and thrombus aspiration, while the ADAPT seems quicker in removing the thrombus. Studies have shown that the ADAPT technique can achieve the same clinical and imaging effects as those achieved by the Solumbra technique in mechanical thrombectomy of LVO (19, 20). However, no studies had compared these two techniques in endovascular management of intracranial atherosclerosis-related LVO. This study was consequently performed to compare the effect and clinical outcomes of the two techniques in recanalization of intracranial atherosclerosis-related LVO in a Chinese cohort.

## Methods

### Subjects

This study was approved by the ethics committee of our hospital, and all patients had given their signed informed consent to participate. Between March 2018 and August 2019, patients with LVO treated with either the Solumbra or ADAPT technique were enrolled. The residual stenosis >70% of a cerebral artery after the first-pass thrombectomy is usually used as a golden criterion to diagnose ICAS-LVO (21–23). If the residual stenosis is below 70%, but its distal blood flow is impaired, or it tends to re-occlude, it is also considered ICAS-LVO. Other indicators of ICAS-LVO may also be required to assist the definitive diagnosis, including truncal-type occlusions or the sign called the “microcatheter first-pass effect,” which can be used to diagnose an ICAS-LVO. The inclusion criteria were patients with LVO, age ≥18 years, the time from disease onset to femoral artery puncture ≤8 h or between 8 and 24 h but consistent with the inclusion criteria of the DAWN experiment or DEFUSE-3 experiment (24), LVO confirmed by computed tomography angiography (CTA) or magnetic resonance angiography (MRA) including occlusion of the intracranial segment of the internal carotid artery (ICA), M1 segment of the middle cerebral artery (MCA), intracranial segments of the vertebral artery and basilar artery, atherosclerotic stenosis-related LVO, the modified Rankin scale score (mRS) ≤2, and baseline score of the National Institutes of Health Stroke Scale (NIHSS) ≥6. The exclusion criteria were intracranial hemorrhage confirmed by CT or MRI and LVO caused by arterial dissection, Moyamoya disease, or arteritic occlusion.

### Treatment Approaches

Before endovascular treatment, patients who were within the time window for intravenous thrombolysis had rt-PA (recombinant tissue plasminogen activator) at a dose of 0.9 mg/kg. After digital subtraction angiography revealed the location and length of LVO, arterial stenosis, and collateral circulation, appropriate endovascular approaches were chosen for treatment. In both the Solumbra and ADAPT techniques, a 300-mm micro-guidewire was used to assist the microcatheter through the occlusion lesion so that a stent or suction catheter could be navigated in place.

### Solumbra Technique

With the Solumbra technique, a long sheath was sent to the distal cervical segment of the ICA or the vertebral artery using an exchange technique under general or local anesthesia, and a 0.025-inch microcatheter (Rebar27, Medtronic Neurovascular, Irvine, CA, USA) harboring a 0.016-inch micro-guidewire was introduced into a large-bore aspiration catheter (ACE, Penumbra, Oakland, CA, USA; or the SOFIA, MicroVention Terumo, Tustin, CA, USA), which were all navigated into the long sheath as a unit. After the 0.025-inch microcatheter was navigated through the thrombus along the micro-guidewire, the large-bore aspiration catheter was sent to the proximal end of the thrombus as close as possible before deployment of the Solitaire FR stent-retriever (Medtronic Neurovascular, Irvine, CA, USA) across the thrombus through the 0.025-inch microcatheter. The microcatheter was then completely removed. Three to five minutes later, the large aspiration catheter was connected for continuous aspiration while advancing the aspiration catheter to the proximal end of the thrombus as close as possible. If recanalization was not successful after aspiration for three times, the DAPT technique would be tried for recanalization.

### DAPT Technique

The establishment of the access road to the thrombus was the same as that in the Solumbra technique. After the aspiration catheter (ACE60, Penumbra, Oakland, CA, USA; or the SOFIA PLUS, MicroVention Terumo, Tustin, CA, USA; REACT68, Medtronic Neurovascular, Irvine, CA, USA) was navigated to the proximal end of the thrombus as close as possible through a 0.021-inch microcatheter (Rebar27, Medtronic Neurovascular, Irvine, CA, USA) harboring a 0.014-inch 300-mm micro-guidewire, it was connected to a negative pressure suction pump for continuous aspiration for 60–90 s. Then, the aspiration catheter was slowly withdrawn until the blood flow velocity returned to normal in the connecting pipe of the negative pressure pump. If the flow velocity did not resume normal, the aspiration catheter was gradually removed under continuous negative pressure. This process was repeated three times until successful reperfusion. If the path is extremely tortuous and the suction catheter cannot be in place, a 300-mm micro-guidewire was used to help the microcatheter through the thrombus-occluded segment. If reperfusion could not be achieved after trying three times, the Solumbra technique was considered.

### Remedial Measures for Treatment

If reperfusion was not successful after the Solumbra or the ADAPT technique had been tried three times, the LVO was thought to be an intractable vessel occlusion, and remedial measures would be taken. If the aspiration catheter could not be navigated through the occlusion segment, a 1.5- or 2-mm diameter rapid-exchanging balloon would be sent to expand the occluded segment while advancing the aspiration catheter, which was connected to the negative pressure pump for thrombus aspiration. The balloon was deflated and withdrawn after the aspiration catheter was in place. This was the so-called balloon-assisted aspiration technique. If recanalization was successful, but the blood flow was obviously limited due to severe vascular stenosis, or there was a tendency of vascular occlusion, remedial measures would be taken including mechanical thrombectomy, percutaneous balloon angioplasty, intracranial stent implantation, and intra-arterial injection of tirofiban (0.5–2.0 mg). For patients with intracranial stent implantation, intravenous tirofiban maintenance therapy was given after the treatment procedure. If the patient did not have intracranial hemorrhage or other hemorrhagic complications, double anticoagulation therapy with aspirin (100 mg/day) and clopidogrel (75 mg/day) was administered the second day after the procedure.

### Effect Evaluation and Follow-Up

The modified Thrombolysis in Cerebral Infarction (mTICI) score was used to evaluate the recanalization of the large vessels during the procedure, and successful recanalization was defined as mTICI 2b-3. Post-procedure CT or MRI was performed to check if intracranial hemorrhage was present, and the intracranial hemorrhage was defined as any intracranial hemorrhage with increased NIHSS score ≥4.

Three months after the procedure, all patients were followed up either through outpatient clinics or *via* telephone contact. The prognosis was assessed with a good prognosis of mRS score between 0 and 2. Three to six months after the procedure, digital subtraction angiography was performed for patients with deployment of intracranial stents for possible in-stent restenosis.

### Statistical Analysis

The statistical analysis was performed with the SPSS 19.0 software (IBM, Chicago, IL, USA). Measurement data in normal distribution were presented as mean ± standard deviation (normal distribution) or median with interquartile range (skewed distribution) and tested with the paired *t*-test or Mann–Whitney *U*-test as indicated between groups. Enumeration data were presented as percentage (*n* and %) with their corresponding 95% confidence intervals (CIs) and tested with the Chi square test. The significant *P* was set at <0.05.

## Results

### Subjects

**Figure 1:**
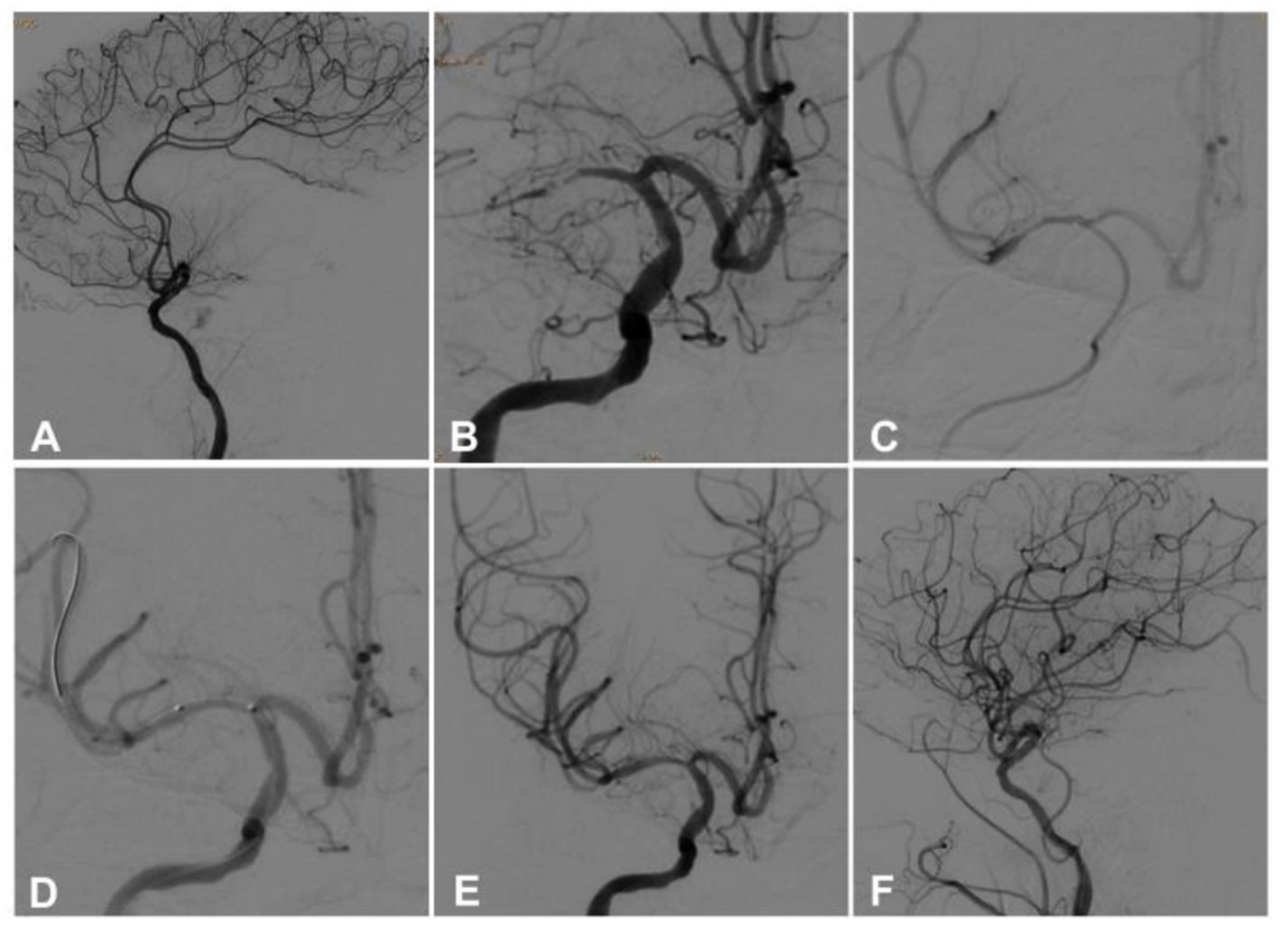
A 36-year-old man had sudden onset of left limb weakness 17 h ago, and the symptom was relieved after intravenous thrombolysis. However, the symptom was aggravated 4 h later, and he was transferred to our hospital with the NIHSS score of 11. Magnetic resonance imaging demonstrated acute infarction in the right basal ganglia. **(A,B)** Before aspiration, cerebral angiography revealed occlusion of the right middle cerebral artery. **(C)** After the thrombus was removed by the aspiration catheter connected to the negative pressure pump, the blood flow forward was resumed. **(D)** During the procedure, a 2 mm × 12 mm balloon was used to expand the stenotic segment. **(E,F)** An Enterprise stent (4.5 mm × 22 mm) was deployed at the stenotic location, and the blood flow was restored to the modified Thrombolysis in Cerebral Infarction (mTICI) grade 3.

**Figure 2:**
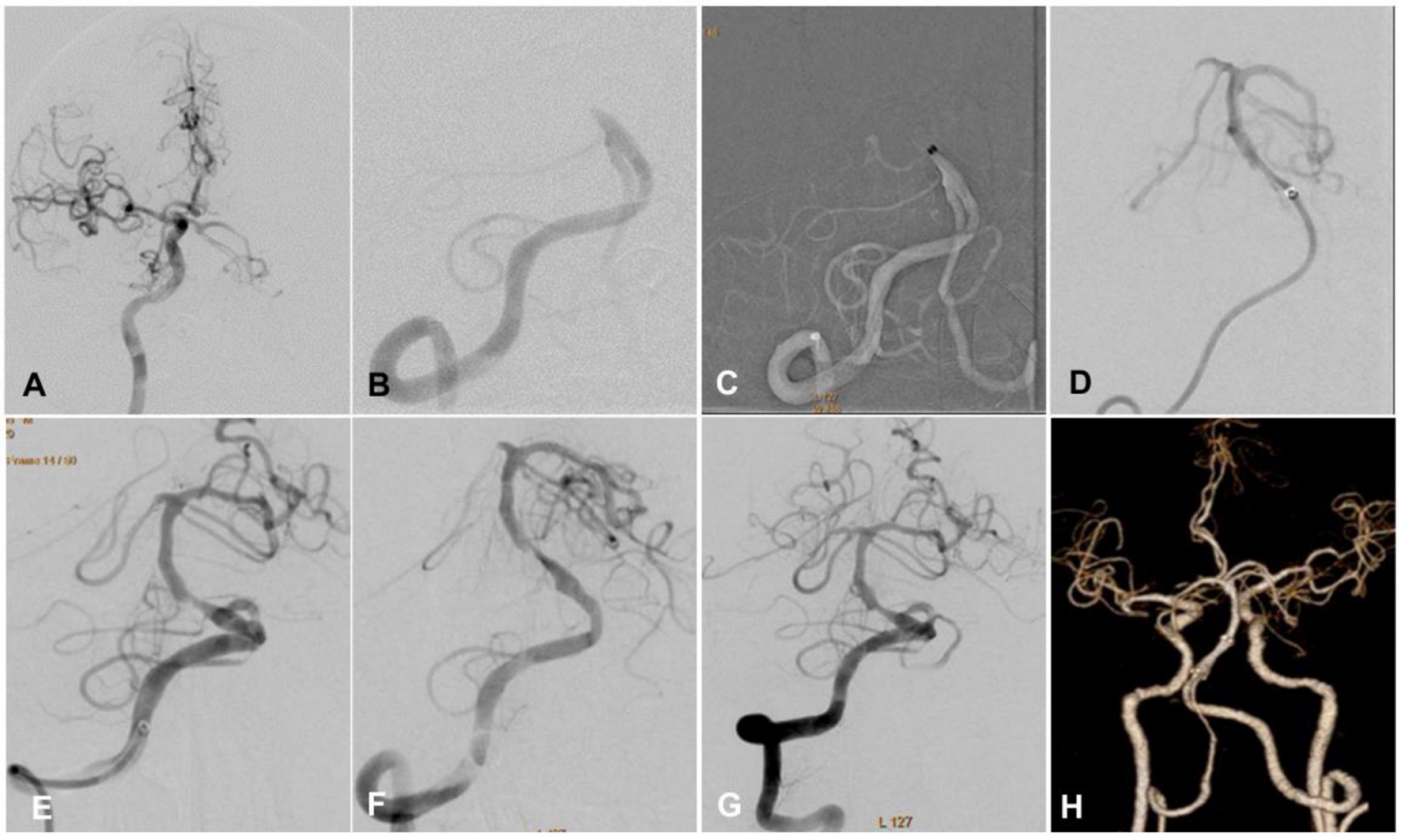
A male patient in his 60s had paroxysmal dysphasia, left limb weakness for 1 h, and coma for 30 min. The NHISS score was 19. **(A)** Cerebral digital subtraction angiography showed lateral circulation and basilar artery tip, suggesting intracranial atherosclerotic stenosis-related large vessel occlusion (ICAS-LVO). **(B)** The right vertebral artery angiography demonstrated occlusion of the initial segment of the basilar artery. **(C)** A REACT68 suction catheter was navigated to the basilar artery proximal to the occlusion, and after 30 s of negative pressure suction *in situ*, the retrograde blood flow in the catheter suddenly recovered. **(D)** Angiography confirmed that the anterior blood flow of the basilar artery returned to normal. **(E)** The aspiration catheter was withdrawn, and angiography revealed severe stenosis at the middle segment of the basilar artery as ICAS-LVO. **(F)** After 10 min of observation, the stenosis degree was increased, and the forward blood flow gradually became worse. **(G)** A stent was implanted after balloon pre-dilation, with the blood flow reaching mTICI grade 3. **(H)** Computed tomography angiography of the head and neck was performed 3 days after operation, which indicated that the stent was unobstructed, and the basilar artery forward blood flow was stable. Seven days after operation, the patient was discharged, with the NIHSS score of 1.

One hundred and four patients met the inclusion criteria and were enrolled including 77 (74.0%) male and 27 female (26.0%) patients with an age range of 36–88 years (mean 64.05 ± 12.10). Seventy-nine (76.0%) patients had hypertension, 23 (22.1%) had diabetes mellitus, 62 (59.6%) had hyperlipidemia, four (3.8%) had atrial fibrillation, and 24 (23.1%) had smoking. Twenty-six (25%) patients had ICA occlusion, 27 (26.0%) had M1 segment occlusion, 23 (22.1%) had occlusion of ICA-L (involving carotid terminus and MCA), 19 (18.3%) had basilar artery occlusion, and 9 (8.7%) had occlusion of both the basilar and vertebral arteries. The baseline NIHSS score was 16.05 ± 4.61. Forty-eight patients were treated with the Solumbra technique, while 56 with the ADAPT (Figures 1, 2), and no significant (*P* > 0.05) differences were found in the age, sex distribution, and risk factors for atherosclerosis, or baseline NIHSS score between the two groups (Table 1). The proportion of patients with ICA-L serial occlusion was significantly (*P* < 0.05) higher in the ADAPT than in the Solumbra group. Among 104 patients, 10 patients (20.83%) in the Solumbra and nine (16.07%) in the ADAPT group had rt-PA intravenous thrombolysis, with no significant (*P* > 0.05) difference in the proportion between the two groups.

**Table 1:** Demography and clinical data.

**Variables**	**Solumbra (*n* = 48)**	**ADAPT (*n* = 56)**	***t*/χ^**2**^**	***P***
Age (years)	64.27 ± 12.17	63.60 ± 11.73	2.613	0.213
Male	37 (77.08%)	40 (71.43%)	0.333	0.576
Hypertension	36 (75.00%)	43 (76.79%)	0.154	0.776
Diabetes mellitus	11 (22.92%)	12 (21.43%)	0.040	1.000
Hyperlipidemia	28 (58.33%)	34 (60.71%)	1.926	0.200
Atrial fibrillation	2 (4.17%)	2 (3.57%)	0.000	1.000
Smoking	12 (25.00%)	12 (21.43%)	0.154	0.776
Baseline NIHSS score	15.83 ± 4.63	16.49 ± 4.60	3.332	0.122
ASPECTS score	8 (7–10)	7 (6–10)	1.691	0.352
**Medications prior to admission**				
Antiplatelets	27 (56.25)	31 (55.35)	0.976	0.451
Anticoagulants	7 (41.3)	10 (41.3)	0.573	0.642
Favorable collaterals	(41.3)	(44.8)	0.663	0.570
**Occluded artery**				
ICA	12 (25.0%)	14 (25.0%)	1.579	0.452
MCA	12 (25.0%)	15 (26.79%)		
ICA-L type	11 (22.92%)	16 (28.57%)		
Basilar artery	9 (18.75%)	10 (17.86%)		
Vertebral terminus	4 (8.33%)	5 (8.93%)		
Intravenous thrombolysis	10 (20.83%)	9 (16.07%)	1.777	0.761

*ICA-L type does not mean a tandem occlusion but occlusion involving carotid terminus and MCA. Vertebral terminus does not mean a tandem occlusion but occlusion involving two anatomical locations of the vertebral terminus and basilar arteries*.

### Clinical Effect and Follow-Up

The mean time from femoral access to recanalization was 76.57 ± 27.23 min for all patients, with a significantly (*P* < 0.05) shorter time for the ADAPT technique than that for the Solumbra (73.61 ± 24.66 vs. 78.05 ± 30.55 min) (Table 2). Twenty-one (43.75%) patients had recanalization at the first pass in the Solumbra group, which was not significantly (*P* > 0.05) different from that in the ADAPT group (*n* = 26, 46.2%). The recanalization time at the first pass was significantly shorter in the ADAPT group than in the Solumbra group (17 ± 10.21 vs. 26 ± 15.55 min, *P* = 0.02). However, the rate of switching to the alternative was significantly higher in the ADAPT group than in the Solumbra group (46.42 vs. 33.33%, *P* = 0.01) (Table 2). Eighty-two patients had eventual recanalization, resulting in a final recanalization rate of 78.85%, with the final recanalization rate of 83.33% (*n* = 40) in the Solumbra group, which was not significantly (*P* > 0.05) different from that in the ADAPT group (75%, *n* = 42).

**Table 2:** Radiologic outcomes of endovascular treatment.

**Variables**	**Solumbra (*n* = 48)**	**ADAPT (*n* = 56)**	**χ^**2**^/*t***	***P***
LKW to puncture time, min	367 (69–721)	302 (74–675)	1.568	0.691
Recanalization by first-pass thrombectomy	12 (25.00%)	9 (16.07%)	0.643	0.553
Recanalization by first-line thrombectomy	21 (43.75%)	26 (46.42%)	0.643	0.553
Time of puncture to recanalization by first-line thrombectomy, min	26 ± 15.55	17 ± 10.21	5.843	0.02
Rescue treatments after first-line thrombectomy Switching to the alternative	16 (33.33%)	26 (46.42%)	2.295	0.01
Tirofiban infusion	39 (81.25%)	49 (87.50%)	0.442	0.863
Balloon angioplasty only	10 (20.83%)	15 (26.78%)	0.651	0.270
Balloon and stenting angioplasty	27 (62.50%)	32 (58.13%)	0.484	0.11
Final successful recanalization	40 (83.33%)	42 (75%)	0.709	0.529
Puncture to final recanalization time, min	78.05 ± 30.55	73.61 ± 24.66	1.121	0.01

*LKW, last known well*.

At 3-month clinical follow-up for all patients (Table 3), the good prognosis rate reached 51.92% with good prognosis in 24 patients (50%) in the Solumbra and 30 (53.57%) in the ADAPT group (Figure 2). The rate of symptomatic intracranial hemorrhage was 14.58% (*n* = 9) in the Solumbra and five 19.64% (*n* = 11) in the ADAPT group. The mortality rate was 21.15% (22/104). No significant (*P* > 0.05) difference existed in the rate of hemorrhagic transformation, parenchymal hemorrhage, subarachnoid hemorrhage, symptomatic intracranial hemorrhage, good prognosis, independent functions, or death (Table 3). Among 80 (76.92%) patients who had angiographic follow-up between 3 and 30 months following endovascular thrombectomy, five (6.25%) patients had in-stent stenosis, one of whom was recanalized with balloon expansion. The other four patients with in-stent stenosis were asymptomatic. Two (2.5%) patients had asymptomatic stent occlusion, which was not managed.

**Table 3:** Clinical outcomes at 3-month follow-up.

**Variables**	**Solumbra (*n* = 48)**	**ADAPT (*n* = 56)**	**χ^**2**^/*t***	***P***
**Hemorrhagic complication**				
HT	12 (25.00%)	16 (28.57%)	0.111	0.806
PH 2	6 (12.50%)	5 (8.93%)	0.571	0.108
SAH	3 (6.25%)	4 (7.1%)	0.177	0.761
sICH	9 (18.75)	11 (19.64)	0.812	0.358
Good functions (mRS 0–2)	24 (50.00%)	30 (53.57%)	0.111	0.806
Independent functions (mRS 0–1)	16 (33.33%)	21 (37.50%)	0.561	0.426
Mortality	10 (20.83)	12 (21.42)	0.623	0.437

*HT, hemorrhagic transformation; PH2, parenchymal hemorrhage, type 2; SAH, subarachnoid hemorrhage; sICH, symptomatic intracranial hemorrhage; mRS, modified Rankin scale score*.

## Discussion

This study investigated the effect and clinical outcomes of the Solumbra and ADAPT technique in treating patients with intracranial atherosclerosis-related LVO, and it was found that the ADAPT technique is significantly quicker to obtain recanalization than the Solumbra technique (73.61 ± 24.66 vs. 78.05 ± 30.55 min), but no significant differences existed in the other aspects including the first-pass recanalization rate, final recanalization rate, periprocedural complication rate, good prognosis rate, and mortality at 3-month follow-up.

After comparing the effect and adverse events of the direct contact aspiration technique vs. the standard stent retriever technique as a first-line endovascular therapy for successful revascularization of acute ischemic stroke and LVO in the anterior circulation, Lapergue et al. (19) found no significant (*P* > 0.05) difference in the recanalization rate in the contact aspiration group vs. the stent retriever group [85.4 vs. 83.1%, odds ratio or OR: 1.20 (95% CI, 0.68–2.10); *P* = 0.53; difference, 2.4% (95% CI, −5.4 to 9.7%)]. No significant difference existed in the clinical efficacy outcomes at 90 days and adverse events. They drew a conclusion that first-line thrombectomy with contact aspiration did not have an increased recanalization rate compared with the stent retriever technique. After studying reperfusion, adverse events, neurological recovery, and functional outcomes of patients with isolated M2 occlusions in 79 patients treated with the contact aspiration or the stent retriever technique, Gory et al. (25) found no significant difference in the reperfusion rate, in the 90-day mRS score (≤ 2) rate (54.4 vs. 50.0%; *P* = 0.84), 24-h change in NIHSS score, or the Alberta Stroke Program Early Computed Tomography score, with the conclusion being that first-line mechanical thrombectomy with contact aspiration vs. stent retriever did not result in an increased successful revascularization rate in patients with acute stroke caused by isolated M2 occlusion. Zhu et al. (26) also found no significant increase in the successful reperfusion rate in acute ischemic stroke patients with LVO of the anterior circulation in the contact aspiration group compared with the stent retriever group. However, in a PRISMA-compliant systematic review and meta-analysis investigating the efficacy and safety of the direct aspiration approach of thrombectomy for recanalization in patients with acute ischemic stroke compared with the stent-retriever approach, Qin et al. (27) found better functional outcomes at 3 months defined as an mRS score of 0 to 2 (OR, 0.77; 95% CI, 0.66–0.97; *P* = 0.03), fewer adverse events especially in symptomatic intracerebral hemorrhage (OR, 0.56; 95% CI, 0.33–0.98; *P* = 0.04) and embolization to a new territory (OR, 0.49; 95% CI, 0.28–0.84; *P* = 0.01) in the direct aspiration group than in the stent retriever group even though no significant differences existed in the rate of successful recanalization between the two groups.

The above studies indicated that the direct contact aspiration technique is as good as or better than the stent retriever technique in achieving the recanalization rate or clinical outcomes in treating patients with LVO. These studies were conducted with only thrombus-related occlusion of LVO without the concomitant factor of atherosclerosis. Thus, the effect of direct contact aspiration with the ADAPT technique vs. the Solumbra technique with combined stent retriever and aspiration is not clear for patients with intracranial atherosclerosis-related LVO. These two entities of disease are different in the clinical presentation, risk factors, and demographic features (8), and the clinical outcomes treated with mechanical thrombectomy may also be different. Baek et al. (7) studied the outcomes of mechanical thrombectomy for acute intracranial LVO and found that recanalization with a stent retriever is significantly (*P* < 0.001) less successful in patients with intracranial atherosclerosis-related LVO, and more patients with intracranial atherosclerosis-related LVO need specific rescue measures including balloon angioplasty, stenting, and intra-arterial glycoprotein IIb/IIIa inhibitor infusion, even though the rates for favorable outcomes, mortality, and symptomatic intracranial hemorrhage were not significantly different between the two groups. In investigating endovascular and clinical outcomes of vertebrobasilar intracranial atherosclerosis-related LVO, Baek et al. (28) also found a similar recanalization rate in patients of LVO with or without concurrent intracranial atherosclerosis, but the recanalization using conventional endovascular modalities such as stent retriever thrombectomy, contact aspiration, or intra-arterial urokinase infusion was less successful in patients with atherosclerosis-related LVO. In our study using the Solumbra technique with the stent retriever combined with aspiration vs. the ADAPT technique of contact aspiration, similar effects and clinical outcomes had been achieved. However, the ADAPT technique was quicker to achieve recanalization than the Solumbra technique, especially when a larger suction catheter is applied, which had been confirmed by some authors (19, 20). The mechanism of suction in the ADAPT technique is to aspirate the whole thrombus without further advancing the catheter once the catheter is in place (29), whereas the Solumbra technique is to send a stent to capture the thrombus and pull it out. The ADAPT technique is thus much quicker than the Solumbra technique in resuming blood flow. Being quicker, cheaper, and comparable in the clinical outcomes and safety, the ADAPT technique has thus become a comparable technique to the standard Solumbra technique in recanalizing intracranial atherosclerosis-related LVO. However, compared with the Solumbra technique, the ADAPT technique has a significantly (*P* < 0.05) greater rate (33.33 vs. 46.42%) of switching to the alternative because atherosclerosis-caused arterial stenosis and local arterial course may readily affect the efficiency of the ADAPT technique compared with the Solumbra technique. Moreover, when the suction catheter is parallel to the long axis of the thrombus in the ADAPT technique, the suction efficiency is the greatest; however, when the suction catheter forms an angle with the long axis of the thrombus (or the artery), the suction efficiency is decreased, and the thrombi may not all be aspirated (30). In the actual situation of suction, it may not always be easy to set the suction catheter parallel to the long axis of thrombus, especially in cases of ICAS-LVO, and it thus needs more remedial measures.

Recently, a study by Yoo et al. investigated the immediate effect of first-line thrombectomy devices for intracranial atherosclerosis-related occlusion using stent retrievers vs. contact aspiration (31). Successful reperfusion was more frequently achieved after first-line thrombectomy in the stent retriever group than in the contact aspiration group (77.6 vs. 43.5%, *P* = 0.001), with significantly fewer remedial measures (12.2 vs. 59.7%, *P* < 0.001) or lower iatrogenic dissection rate (8.2 vs. 29.0%, *P* = 0.012) in the stent retriever group. After remedial measures, the final successful reperfusion rate was similar in both groups (87.8 vs. 77.4%, *P* = 0.247), with no significant difference in the 3-month good outcomes (modified Rankin Scale, *P* = 0.524). However, this study was performed between 2011 and 2016 when the first-generation aspiration devices were used, while no aspiration-assisted techniques were applied in the stent retriever group. Currently, the second-generation aspiration devices with larger-diameter suction catheters had been applied and had achieved a better reperfusion rate and good prognosis rate. Moreover, the Solumbra technique had been standardized. In our study, the latest devices were used, and to compare the efficacy of these two mainstream techniques in ICAS-LVO cases may have more clinical significance.

Acute intracranial atherosclerosis-related LVO may be caused by thrombi at the stenotic location, at the segment proximal or distal to the stenosis, or covering a longer segment both proximal and distal to the stenosis. When the thrombus is only at the stenotic location, the thrombus load is small or soft with high permeability. In this situation, application of the ADAPT technique can easily remove the thrombus without damaging the intima at the stenosis caused by use of the Solumbra technique. Some authors suggested the use of balloon angioplasty for thrombus at the stenotic location (32). However, direct balloon angioplasty may cause distal embolization by escaped thrombi, injury to perforating arteries, and possible escape of the balloon or stent. For thrombi that are located at the segment only proximal to the stenosis or both proximal and distal to the stenosis, the current focus is on the management of the thrombus at the segment distal to the stenosis using the ADAPT technique. Because the aspiration catheter, if located proximal to the stenosis, has a low efficiency for aspirating thrombi distal to the stenosis, it is thus necessary to dilate the stenotic segment before navigating the aspiration catheter beyond the stenosis for aspiration. However, in doing this, the risk of thrombus escape is increased. Previous studies have demonstrated that stent pulling at the stenotic segment will damage the intima and that the stenosis will also increase the power of the stent to cut the thrombus, leading to the distal escape of more thrombi (33, 34). In our study using the ADAPT technique, we used an assisted aspiration technique as a rescue treatment in a small number of cases. A small-diameter balloon (1.5 or 2 mm) was used to predilate the stenotic segment while applying negative pressure for continuous aspiration, which effectively prevented thrombus escape distally. This is primarily based on the fact that the tip of the aspiration catheter has a 2-mm external diameter, and that after predilation with a 2-mm balloon, the passage of the aspiration catheter through the stenotic segment can limit forward flow, facilitating negative pressure aspiration of distal thrombus. Moreover, the ADAPT technique is the commonly used technique in clinics, and it has been confirmed that the aspiration catheter will not increase the risk of distal thrombus escape when passing through the stenotic segment (35). Third, a 2-mm rapid exchange balloon along a 300-mm intracranial support wire can be quickly navigated to the required location or withdrawn without increasing the operation time. Of course, if the occlusion is located in an arterial segment rich in perforating vessels, the risk of perforator occlusion will be increased if balloon dilation is performed before removal of the high-load thrombus.

Recently, the ADAPT technique has gradually gained clinical acceptance primarily because of the invention of large-caliber catheters suitable for thrombus aspiration and improvement of direct aspiration technology. The latest generation of large-diameter aspiration catheter can ensure enough local negative pressure for suction of thrombus and has excellent flexibility for passing through tortuous blood vessels to reach the required location, being more effective especially for cardiogenic or arterial emboli with large load. However, the key to the ADAPT technique is to ensure sufficient local negative pressure for aspiration. For intracranial atherosclerosis-related LVO with tortuous vessels or long segment stenosis, the effect of aspiration and efficiency will be affected even with balloon predilation because the large caliber catheter still has the risk of cutting plaques, causing vasospasm or even arterial dissection when passing through the stenosis. Tortuous vessels and long-segment stenosis may direct the negative pressure toward the vascular wall, affecting the treatment effect and efficiency.

Some limitations existed in this study including the retrospective nature, single center study, a small cohort of patients, and the enrolled Chinese patients only. Moreover, some patients had intravenous thrombolysis before enrollment, which may affect the effect of mechanical thrombectomy. All these factors may affect the generalization of the outcome of this study. Future studies will have to resolve these issues for better clinical outcomes.

## Conclusion

In conclusion, in patients with acute intracranial atherosclerosis-related LVO, clinical outcomes and safety treated with the ADAPT technique are comparable with those achieved with the Solumbra technique even though additional remedial measures are required for both groups for complete recanalization.

## Data Availability Statement

The raw data supporting the conclusions of this article will be made available by the authors, without undue reservation.

## Ethics Statement

The studies involving human participants were reviewed and approved by Ethics committee of Henan Provincial People's Hospital, Henan university. The patients/participants provided their written informed consent to participate in this study.

## Author Contributions

Z-SL, T-FZ, QL, L-FZ, and Z-LW collected the data. Z-SL, T-XL, and B-LG designed the study. Z-SL and B-LG analyzed the data. L-FZ supervised the study. Z-SL wrote the original article. B-LG revised the article. All authors have read and approved the manuscript.

## Conflict of Interest

The authors declare that the research was conducted in the absence of any commercial or financial relationships that could be construed as a potential conflict of interest.

## Abbreviations

LVO: large vessel occlusion
ADAPT: A Direct Aspiration First-Pass Thrombectomy
CTA: computed tomography angiography
MRA: r magnetic resonance angiography
ICA: intracranial segment of the internal carotid artery
MCA: middle cerebral artery
mRS: modified Rankin scale score
NIHSS: National Institutes of Health Stroke Scale.

## References

[1] Rha JH Saver JL. The impact of recanalization on ischemic stroke outcome: a meta-analysis. Stroke. (2007) 38:967–73. 10.1161/01.STR.0000258112.14918.24 17272772

[2] Turk AS Spiotta A Frei D Mocco J Baxter B Fiorella D. Initial clinical experience with the adapt technique: a direct aspiration first pass technique for stroke thrombectomy. J Neurointerv Surg. (2018) 10:i20–5. 10.1136/neurintsurg-2013-010713.rep 30037948

[3] Goyal M Menon BK van Zwam WH Dippel DW Mitchell PJ Demchuk AM. Endovascular thrombectomy after large-vessel ischaemic stroke: a meta-analysis of individual patient data from five randomised trials. Lancet. (2016) 387:1723–31. 10.1016/S0140-6736(16)00163-X 26898852

[4] Hong KS Ko SB Yu KH Jung C Park SQ Kim BM. Update of the Korean clinical practice guidelines for endovascular recanalization therapy in patients with acute ischemic stroke. J Stroke. (2016) 18:102–13. 10.5853/jos.2015.01655 26846761 PMC4747068

[5] Powers WJ Derdeyn CP Biller J Coffey CS Hoh BL Jauch EC. 2015 American heart association/American stroke association focused update of the 2013 guidelines for the early management of patients with acute ischemic stroke regarding endovascular treatment: a guideline for healthcare professionals from the American Heart Association/American Stroke Association. Stroke. (2015) 46:3020–35. 10.1161/STR.0000000000000074 26123479

[6] Powers WJ Rabinstein AA Ackerson T Adeoye OM Bambakidis NC Becker K. 2018 guidelines for the early management of patients with acute ischemic stroke: A guideline for healthcare professionals from the American Heart Association/American Stroke Association. Stroke. (2018) 49:e46–110. 10.1161/STR.0000000000000172 29367334

[7] Kang DH Jung C Yoon W Kim SK Baek BH Kim JT. Endovascular thrombectomy for acute basilar artery occlusion: a multicenter retrospective observational study. J Am Heart Assoc. (2018) 7:e009419. 10.1161/JAHA.118.009419 29982231 PMC6064858

[8] Yang W Zhang Y Li Z Zhang L Li H Hua W. Differences in safety and efficacy of endovascular treatment for acute ischemic stroke: a propensity score analysis of intracranial atherosclerosis-related occlusion versus embolism. Clin Neuroradiol. (2020). 10.1007/s00062-020-00899-x. [Epub ahead of print]. 32239261

[9] Baek JH Kim BM Kim DJ Heo JH Nam HS Song D. Importance of truncal-type occlusion in stentriever-based thrombectomy for acute stroke. Neurology. (2016) 87:1542–50. 10.1212/WNL.0000000000003202 27629085

[10] Lee JS Hong JM Lee KS Suh HI Choi JW Kim SY. Primary stent retrieval for acute intracranial large artery occlusion due to atherosclerotic disease. J Stroke. (2016) 18:96–101. 10.5853/jos.2015.01347 26467196 PMC4747073

[11] Matias-Guiu JA Serna-Candel C Matias-Guiu J. Stroke etiology determines effectiveness of retrievable stents. J Neurointerv Surg. (2014) 6:e11. 10.1136/neurintsurg-2012-010395 22591732

[12] Al Kasab S Almadidy Z Spiotta AM Turk AS Chaudry MI Hungerford JP. Endovascular treatment for AIS with underlying ICAD. J Neurointerv Surg. (2017) 9:948–51. 10.1136/neurintsurg-2016-012529 27502403

[13] Jia B Feng L Liebeskind DS Huo X Gao F Ma N. Mechanical thrombectomy and rescue therapy for intracranial large artery occlusion with underlying atherosclerosis. J Neurointerv Surg. (2018) 10:746–50. 10.1136/neurintsurg-2017-013489 29203731

[14] Delgado Almandoz JE Kayan Y Young ML Fease JL Scholz JM Milner AM. Comparison of clinical outcomes in patients with acute ischemic strokes treated with mechanical thrombectomy using either solumbra or adapt techniques. J Neurointerv Surg. (2016) 8:1123–8. 10.1136/neurintsurg-2015-012122 26667250

[15] Prochazka V Jonszta T Czerny D Krajca J Roubec M Hurtikova E. Comparison of mechanical thrombectomy with contact aspiration, stent retriever, and combined procedures in patients with large-vessel occlusion in acute ischemic stroke. Med Sci Monit. (2018) 24:9342–53. 10.12659/MSM.913458 30578729 PMC6320656

[16] Deshaies EM. Tri-axial system using the solitaire-fr and penumbra aspiration microcatheter for acute mechanical thrombectomy. J Clin Neurosci. (2013) 20:1303–5. 10.1016/j.jocn.2012.10.037 23835465

[17] Humphries W Hoit D Doss VT Elijovich L Frei D Loy D. Distal aspiration with retrievable stent assisted thrombectomy for the treatment of acute ischemic stroke. J Neurointerv Surg. (2015) 7:90–4. 10.1136/neurintsurg-2013-010986 24463439

[18] Lee JS Hong JM Lee SJ Joo IS Lim YC Kim SY. The combined use of mechanical thrombectomy devices is feasible for treating acute carotid terminus occlusion. Acta Neurochir (Wien). (2013) 155:635–41. 10.1007/s00701-013-1649-5 23435866

[19] Lapergue B Blanc R Gory B Labreuche J Duhamel A Marnat G. Effect of endovascular contact aspiration vs. stent retriever on revascularization in patients with acute ischemic stroke and large vessel occlusion: the aster randomized clinical trial. JAMA. (2017) 318:443–52. 10.1001/jama.2017.9644 28763550 PMC5817613

[20] Turk AS 3rd Siddiqui A Fifi JT De Leacy RA Fiorella DJ Gu E. Aspiration thrombectomy versus stent retriever thrombectomy as first-line approach for large vessel occlusion (compass): a multicentre, randomised, open label, blinded outcome, non-inferiority trial. Lancet. (2019) 393:998–1008. 10.1016/S0140-6736(19)30297-1 30860055

[21] Lee JS Lee S-J Yoo JS Hong JH Kim CH Kim YW. Prognosis of acute intracranial atherosclerosis-related occlusion after endovascular treatment. J Stroke. (2018) 20:394–403. 10.5853/jos.2018.01627 30309234 PMC6186924

[22] Qureshi AI Caplan LR. Intracranial atherosclerosis. Lancet. (2014) 383:984–98. 10.1016/S0140-6736(13)61088-0 24007975

[23] Tsang ACO Lau KK Tsang FCP Tse MMY Lee R Lui WM. Severity of intracranial carotid artery calcification in intracranial atherosclerosis-related occlusion treated with endovascular thrombectomy. Clin Neurol Neurosurg. (2018) 174:214–6. 10.1016/j.clineuro.2018.09.030 30278297

[24] Ragoschke-Schumm A Walter S. Dawn and defuse-3 trials: is time still important? Radiologe. (2018) 58:20–3. 10.1007/s00117-018-0406-4 29808241

[25] Gory B Lapergue B Blanc R Labreuche J Ben Machaa M Duhamel A. Contact aspiration versus stent retriever in patients with acute ischemic stroke with m2 occlusion in the aster randomized trial (contact aspiration versus stent retriever for successful revascularization). Stroke. (2018) 49:461–4. 10.1161/STROKEAHA.117.019598 29284735

[26] Zhu F Lapergue B Kyheng M Blanc R Labreuche J Ben Machaa M. Similar outcomes for contact aspiration and stent retriever use according to the admission clot burden score in aster. Stroke. (2018) 49:1669–77. 10.1161/STROKEAHA.118.021120 29880554

[27] Qin C Shang K Xu SB Wang W Zhang Q Tian DS. Efficacy and safety of direct aspiration versus stent-retriever for recanalization in acute cerebral infarction: a prisma-compliant systematic review and meta-analysis. Medicine (Baltimore). (2018) 97:e12770. 10.1097/MD.0000000000012770 30313091 PMC6203566

[28] Baek JH Kim BM Heo JH Kim DJ Nam HS Kim YD. Endovascular and clinical outcomes of vertebrobasilar intracranial atherosclerosis-related large vessel occlusion. Front Neurol. (2019) 10:215. 10.3389/fneur.2019.00215 30941084 PMC6433872

[29] Alawieh A Chatterjee AR Vargas J Chaudry MI Lena J Turner R. Lessons learned over more than 500 stroke thrombectomies using adapt with increasing aspiration catheter size. Neurosurgery. (2020) 86:61–70. 10.1093/neuros/nyy444 30418596

[30] Bernava G Rosi A Boto J Brina O Kulcsar Z Czarnetzki C. Direct thromboaspiration efficacy for mechanical thrombectomy is related to the angle of interaction between the aspiration catheter and the clot. J Neurointerv Surg. (2020) 12:396–400. 10.1136/neurintsurg-2019-015113 31548213 PMC7146918

[31] Yoo J Lee S-J Hong J-H Kim Y-W Hong JM Kim C-H. Immediate effects of first-line thrombectomy devices for intracranial atherosclerosis-related occlusion: stent retriever versus contact aspiration. BMC Neurol. (2020) 20:283. 10.1186/s12883-020-01862-6 32682406 PMC7368707

[32] Yi TY Chen WH Wu YM Zhang MF Zhan AL Chen YH. Microcatheter “first-pass effect” predicts acute intracranial artery atherosclerotic disease-related occlusion. Neurosurgery. (2019) 84:1296–305. 10.1093/neuros/nyy183 29790969

[33] Kim SK Yoon W Heo TW Park MS Kang HK. Negative susceptibility vessel sign and underlying intracranial atherosclerotic stenosis in acute middle cerebral artery occlusion. AJNR Am J Neuroradiol. (2015) 36:1266–71. 10.3174/ajnr.A4280 25814657 PMC7965267

[34] Lee JS Hong JM Kim JS. Diagnostic and therapeutic strategies for acute intracranial atherosclerosis-related occlusions. J Stroke. (2017) 19:143–51. 10.5853/jos.2017.00626 28592778 PMC5466291

[35] Gurkas E Akpinar CK Aytac E. Advance: an effective and feasible technique in acute stroke treatment. Interv Neuroradiol. (2017) 23:166–72. 10.1177/1591019916682358 28304200 PMC5433605

